# Establishing the Basis for Mechanobiology-Based Physical Therapy Protocols to Potentiate Cellular Healing and Tissue Regeneration

**DOI:** 10.3389/fphys.2017.00303

**Published:** 2017-06-06

**Authors:** Joanna L. Ng, Mariana E. Kersh, Sharon Kilbreath, M. Knothe Tate

**Affiliations:** ^1^Graduate School of Biomedical Engineering, University of New South WalesSydney, NSW, Australia; ^2^Department of Mechanical Science and Engineering, University of Illinois at Urbana-ChampaignChampaign, IL, United States; ^3^Faculty of Health Sciences, University of SydneySydney, NSW, Australia

**Keywords:** mechanobiology, mechanotransduction, physical therapy, rehabilitation, exercise therapy, tissue regeneration, human health and disease, multiscale adaptation

## Abstract

Life is mechanobiological: mechanical stimuli play a pivotal role in the formation of structurally and functionally appropriate body templates through mechanobiologically-driven cellular and tissue re/modeling. The body responds to mechanical stimuli engendered through physical movement in an integrated fashion, internalizing and transferring forces from organ, through tissue and cellular length scales. In the context of rehabilitation and therapeutic outcomes, such mechanical stimuli are referred to as mechanotherapy. Physical therapists use mechanotherapy and mechanical interventions, e.g., exercise therapy and manual mobilizations, to restore function and treat disease and/or injury. While the effect of directed movement, such as in physical therapy, is well documented at the length scale of the body and its organs, a number of recent studies implicate its integral effect in modulating cellular behavior and subsequent tissue adaptation. Yet the link between movement biomechanics, physical therapy, and subsequent cellular and tissue mechanoadaptation is not well established in the literature. Here we review mechanoadaptation in the context of physical therapy, from organ to cell scale mechanotransduction and cell to organ scale extracellular matrix genesis and re/modeling. We suggest that physical therapy can be developed to harness the mechanosensitivity of cells and tissues, enabling prescriptive definition of physical and mechanical interventions to enhance tissue genesis, healing, and rehabilitation.

## Introduction

**Figure 1:**
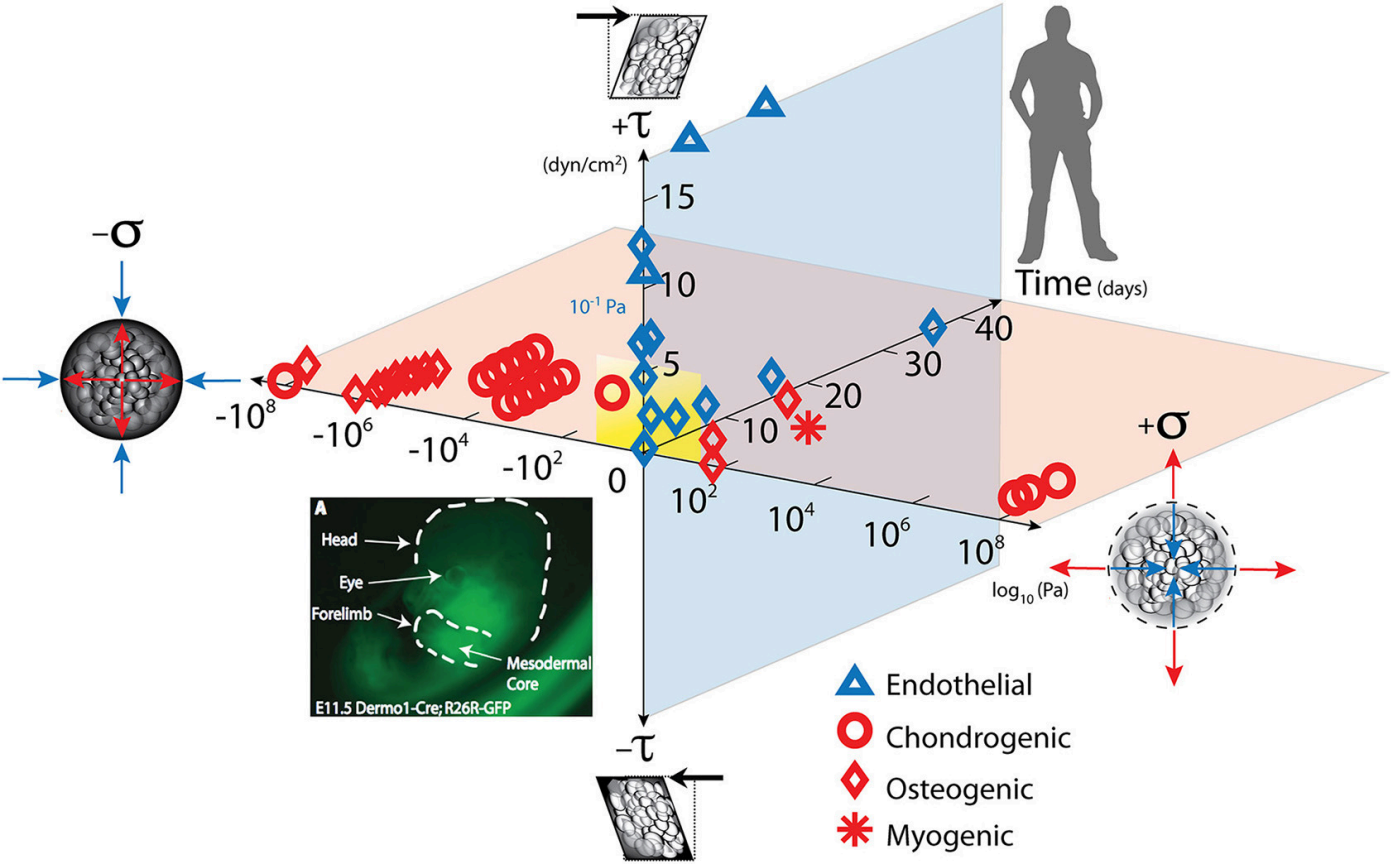
Mechanical loading throughout life literally shapes the structure and function of cells inhabiting the human body. Cells sense mechano-chemical stimuli and prototype tissue templates (modulate tissue genesis, an important aspect of re-/modeling) via up and downregulation of structural protein transcription, secretion into the extracellular matrix, and post-translational modification. This figure depicts characteristic magnitudes and time domains of mechanical signals applied in studies of multipotent cell differentiation, with nascent lineage commitment depicted by the shape of the data points. Tissue genesis and adaptation represents a continuum in space and time, over the life cycle of the individual organism, from development of the body template *in utero* (depicted 11.5 days after fertilization at the first stages of skeletogenesis in the mouse) and in the adult human. Dilatational (volume changing, x-axis, on log scale) and deviatoric (shape changing, y-axis) stresses to which cells are exposed over time (z-axis), modulate lineage commitment (shape of data points indicate fate of stem cells exposed “xyz, stress over time”) and tissue genesis throughout life. Image adapted from Song et al. (2013), Anderson and Knothe Tate (2007), used with permission and available online at https://doi.org/10.1371/journal.pone.0043601.g001.

**Figure 2:**
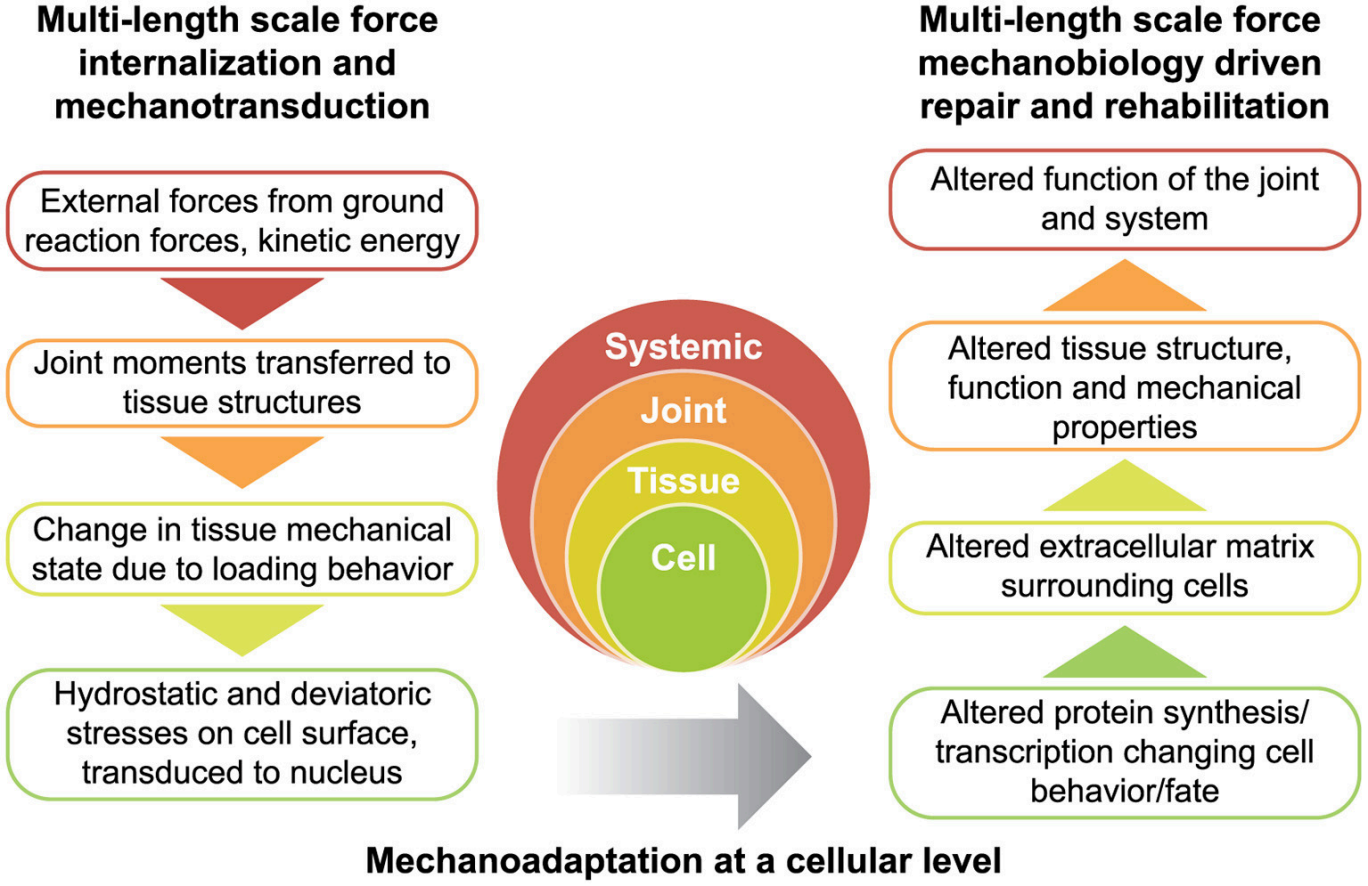
Rationale for physical therapy protocols for mechanotherapy. Force is transferred across multiple length scales (left) while tissues adapt to the dynamic mechanical environment (right). Together, the transfer of force from the environment, and subsequent structure-function adaptation of the system constitute the dynamic process of functional adaptation, also referred to as mechanoadaptation.

Exogenous (outside—in, e.g., ground reaction forces between the outside world and the sole of the foot) and endogenous (inside—out, e.g., heart pumping inside the chest cavity) mechanical stimuli literally shape the body's unfolding template during development, as well during growth and re-/modeling of the body's tissues and organs throughout life (Figure 1)^1^. These forces, which may emanate from a single source such as where the sole of the foot touches the ground, propagate through the body via intersegmental force distribution and dynamic coupling. In this way, the body can be viewed as an interconnected system that responds both locally and globally to mechanical stimuli (Figure 2). Mechanobiology examines how biological tissues are not only shaped by mechanical stimuli but also how tissue re-/modeling via cells actively modulates the mechanical environment of cells themselves (Tate and Niederer, 1998; Knothe Tate et al., 2016a). These processes are known to occur via mechanotransduction and mechanoadaptation, where mechanical stimuli effectively direct tissue re/modeling via cells (Eyckmans et al., 2011; Knothe Tate, 2011; Knothe Tate et al., 2011), yet the Laws of Mechanobiology have yet to be defined.

Physical movement and/or mechanical interventions propagate mechanical stimuli from the length scale of the organismal system (body) to its cellular inhabitants. Mechanotherapy refers to such mechanical stimuli in the context of promoting healing, tissue repair and rehabilitation, i.e., “any intervention that introduces mechanical forces with the goal of altering molecular pathways and inducing a cellular response that enhances tissue growth, modeling, remodeling, and/or repair” (Thompson et al., 2016). Physical therapy, also referred to as physiotherapy, uses physical means to restore function and is considered a form of mechanotherapy.

In this review, we aim to bring forward the knowledge of mechanobiology in context of organismal mechanophysiology, demonstrating the integration of mechanoadaptation and re/modeling across all length scales and establishing the basis for mechanobiology-based physical therapy protocols to potentiate cellular healing and tissue regeneration. Beginning first with a critical overview of mechanical interventions used in physical therapy, this narrative review (level of evidence: 5) focuses primarily on force transfer and mechanoadaptation of musculoskeletal tissues, before describing recent examples of mechanotherapy-based interventions and future directions in the field.

## Linking the physical therapist to joint biomechanics

Physical therapists use exercise therapy and manual therapy to restore function and reduce pain to treat disease and/or injury. Although physical therapy implies the use of physical approaches or movements to treat the body, the opportunity to foster an interactive discourse between human biomechanics and physical therapy has not yet been realized. In a Pubmed “Title/Abstract” search where the terms “biomechanics” and “physical therapy” was entered, there was evidence supporting the importance of exercise therapy and mobilizations to joint rehabilitation, but incomplete evidence linking biomechanics theory to therapeutic outcomes. To bridge the gap between engineering biomechanics and clinical physical therapy, here we describe physical movement in the context of joint biomechanics and draw upon the literature to demonstrate how force distribution in the body can be altered through exercise and/or manual therapy. The aim is to facilitate healing and/or target specific mechanoadaptations to foster healthy balance of forces to prevent future injuries and maximize wellbeing. While the former is specific to physical therapy in a clinical context, the latter expands well beyond physical therapy to exercise physiology and its health benefits throughout life (Warden et al., 2014).

### Physical therapy in the context of joint biomechanics

The use of physical therapy to treat joint diseases and musculoskeletal conditions is well established and documented in the literature. Common practices employed in the treatment of joint diseases, such as osteoarthritis and rheumatoid arthritis, involve the mobilization of joints through exercise therapy (strength training, range of motion exercises, and aerobic activity) and manual therapy (passive physiologic and accessory joint movements) (Jansen et al., 2011). Previous studies have shown that these activities are beneficial in restoring function (Lamb et al., 2015), improving physical activity (Abbott et al., 2013), reducing pain (Jansen et al., 2011), and increasing patient satisfaction (French et al., 2013). These rehabilitation modalities can be viewed in terms of engineering principles, whereby joint mobilizations, such as range of motion exercises and joint movements, can be likened to joint loading and torques in biomechanics (Wardrope et al., 2008). Hence, an understanding of the relationship between movement and torque highlights the possible ways that physical therapy can be used to direct and manipulate forces affecting the body at systemic length scales.

**Figure 3:**
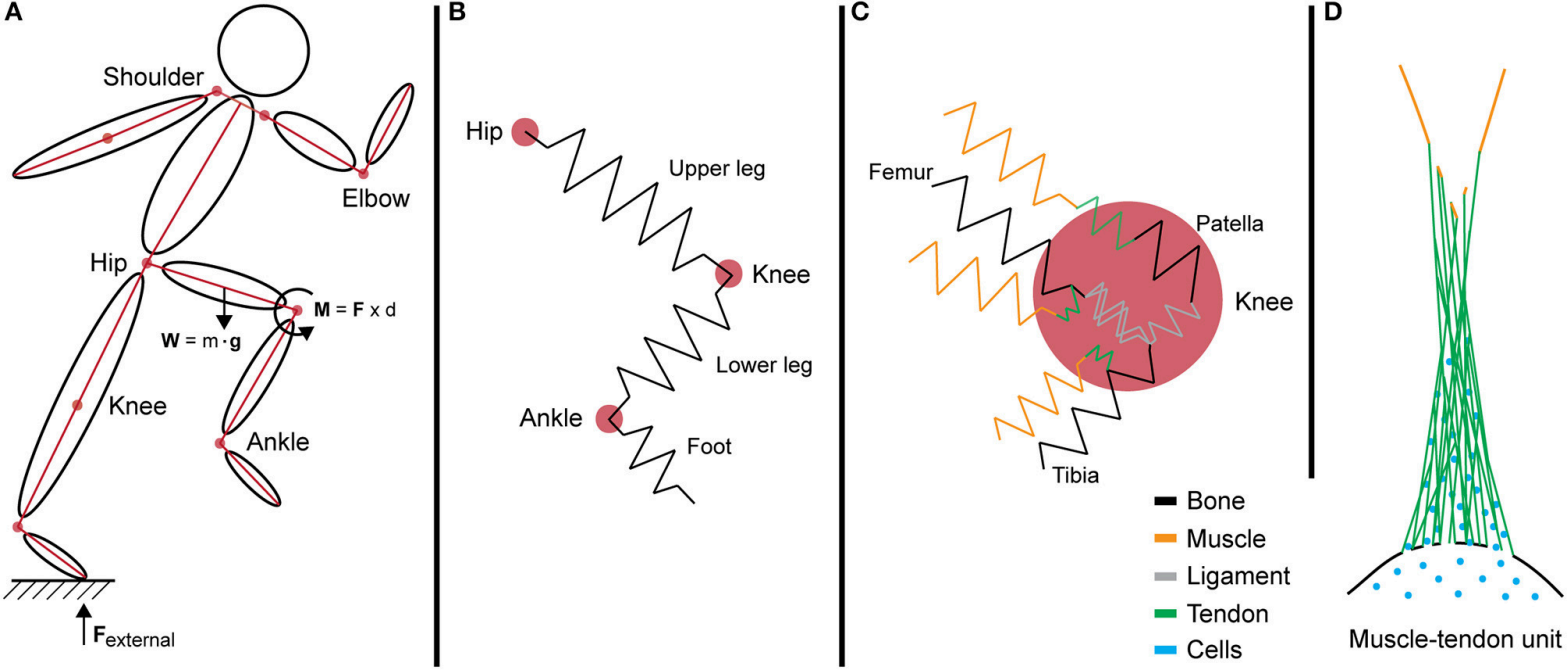
Internalization of forces generated during motion across multi-length scales. **(A)** A simple segment link model depicting the force vectors and joint torque generated during movement at the systemic level. **(B)** In this model, limbs are idealized as springs that resist loading from intersegmental forces. **(C)** The interaction of multiple tissues at the knee joint increases the complexity of the model. Different tissues exhibit varied mechanical properties and stiffness, represented by multi-colored springs. **(D)** Schematic of the muscle—tendon unit at the tissue level showing the anatomical complexities of biological tissues as well as the natural gradients in structure (e.g., composition and architecture) and function (e.g., mechanical stiffness and gradients in force transfer to prevent stress concentration and associated structural weakness).

All physical movements impart forces and torques on the body. Using the idea of “the body as a machine” (Nicholls and Gibson, 2010), the human body can be portrayed as a whole system that moves according to imposed stresses and strains (Wardrope et al., 2008). From a biomechanics perspective, these movements or positional changes of the system are dictated by forces defined by Newton's 2nd Law, where force equals mass times acceleration (*F* = *ma*). The forces involved with motion are transferred through the body via rotational and translational dynamics, which generate moments about joints, i.e., joint torques (Figures 3A,B). Thus, joint torques are a resultant moment of ground reaction force vectors, joint angles, and angular velocity (Wardrope et al., 2008; Farrokhi et al., 2013). Extending on the theme of the body as an integrated machine, changes in torque across one joint are compensated and balanced by the force vectors and moments in other joints (Block and Shakoor, 2010; Yaari et al., 2015). Hence, joint torques cannot be viewed in isolation but as a part of the whole system.

**Figure 4:**
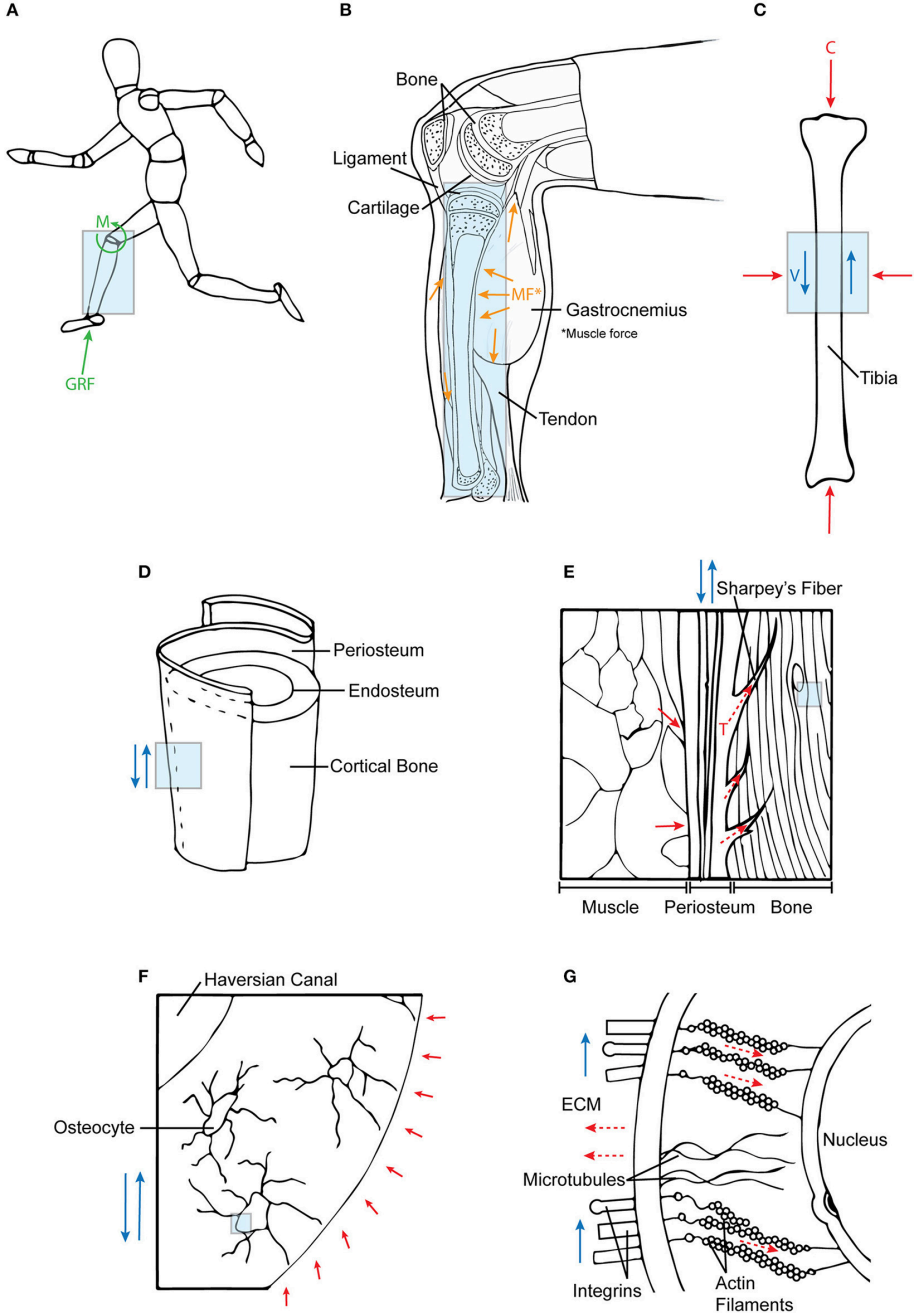
Mechanotransduction of forces across multiple length scales. **(A)** Ground reaction forces and joint moments at the systemic level are **(B)** internalized into tissue structures. In addition, these forces are supported by muscle co-contractions, which **(C)** together result in compressive (C) and fluid flow shear (V) forces acting on bone. **(D,E)** At a tissue level, these forces are transferred to bone via periosteal Sharpey's fibers and muscle attachments. **(F,G)** At the cellular length scale, fluid shear (deviatoric) forces and hydrostatic forces in the ECM affect the cytoskeletal tension (tensegrity) of the cell. Each subsequent diagram is an enlarged view of the previous; the arrows are indicative of force directionality. Green arrows represent external forces before internalization; orange arrows represent forces imparted from muscle; blue arrows depict shear forces; red arrows show compression forces; and dotted red arrows refer to tensile forces.

The joint torque represents the total force that is passing through the joint via intersegmental forces and dynamic coupling. In an ideal model, the sum of all torques is calculated by adding the forces exerted and absorbed by structures interacting at the joint, degrees of freedom, and angular velocities. However, the human body is a complex system with multiple components of varying mechanical properties, and as a whole is beyond the capacity of current biomechanical models. To simplify the mechanics and determine the kinematics of the system, these forces are often depicted in link-segment models. For example, in a link-segment model of the upper and lower leg, the forces acting through these two directional segments create a moment about the knee joint (Figures 3, 4A). This joint torque, as a function of joint angle and angular velocity, is representative of the intersegmental forces and dynamic coupling occurring across the knee (Fleming et al., 2005; Anderson et al., 2007).

### Joint biomechanics interventions for therapeutic outcomes

#### Exercise therapy

Five published studies to date link biomechanics theory directly to clinical physical therapy outcomes (Shepherd and Carr, 1994; Fleming et al., 2005; Block and Shakoor, 2010; Farrokhi et al., 2013; Yaari et al., 2015). These studies show evidence of altered joint physiology as a result of physical therapy intervention in cases of osteoarthritis (Block and Shakoor, 2010; Farrokhi et al., 2013), anterior cruciate ligament reconstruction (Fleming et al., 2005) and total knee arthroplasty (Yaari et al., 2015). Knee osteoarthritis often presents with high compressive loads on the medial tibiofemoral compartment of the knee joint. This is marked by an increased knee adduction moment and a malaligned ground reaction force that passes medial to the knee joint's center of rotation (Farrokhi et al., 2013).

While exercise therapy currently provides a keystone for conservative management of joint disease including osteoarthritis, no evidence-based protocols exist for its practice. The most efficacious exercise modality has not been delineated, and no side-by-side comparisons of efficacy, e.g., between open chain and closed chain kinetic exercises, exist in the literature (Fleming et al., 2005; Page et al., 2011). A consensus for the importance of exercise therapy in joint rehabilitation suggests a pivotal role of increased muscle strength. Since exercise reduces joint loading by increasing muscle strength, and since the muscles crossing the joint are the greatest absorbers of torque, it is postulated that increased muscle strength alleviates osteoarthritic pain and physical disability (Page et al., 2011). Furthermore, through joint compensation by extension of the link-segment model, it has been demonstrated that strengthening hip adductor muscles has practicality in supporting reduced knee medial compartment loading in osteoarthritic patients (Block and Shakoor, 2010). Also, studies have shown that biomechanical alterations, such as the use of taping, bracing, walking aids, and insoles in conjunction with exercise are capable of offloading the injured or diseased joint (Page et al., 2011; Farrokhi et al., 2013; Yaari et al., 2015). For example in osteoarthritic treatment, valgus bracing and lateral heel wedge insoles exhibit the capacity to bring the knee center of rotation in alignment with the ground reaction force line of action and decrease knee adduction moment by 13% during the gait cycle, respectively (Farrokhi et al., 2013; Yaari et al., 2015). Such examples show that biomechanical interventions, in conjunction with exercise, exert a sizable impact on joint rehabilitation and limiting disease progression.

#### Manual therapy

Manual therapy, a hands-on technique involving mobilization and/or manipulation of joints, limbs, and other body parts, represents another form of biomechanical intervention. Mobilization refers to repetitive passive moments of low velocity and varying amplitudes, and manipulation describes high velocity, high force movements at the end of the range of motion (French, 2007; Page et al., 2011). A lack of standardized, efficacy based protocols presents a current hurdle to development of manual therapy as a mechanobiology based physical therapy approach.

Previous studies report that manual physical therapy for the treatment of knee (Jansen et al., 2011; Tragord et al., 2013), hip (Abbott et al., 2013; French et al., 2013), and glenohumeral (Crowell and Tragord, 2015) osteoarthritis alleviates joint pain, improves range of motion and physiological function (Jansen et al., 2011). Interestingly, in studies comparing with current standards of care the efficacy of manual therapy, exercise therapy or both modalities together (Jansen et al., 2011; Abbott et al., 2013; French et al., 2013), implementation of manual therapy showed significant benefit compared to current standards of care. Combining exercise therapy with manual therapy resulted in no perceived or measurable benefit aside from an increase in patient satisfaction. This is perhaps due to the capacity of physical therapists to mobilize the joint directly through anatomical joint movements (e.g., flexion and extension) and stretching of corresponding muscle groups (i.e., flexors and extensors). Hence, the standard of care for manual therapy depends highly on the movements and mobilization forces imparted by the physical therapist.

In a study that quantified force, frequency, dosage (time-force interval) and amplitude of joint mobilization amongst clinicians treating knee osteoarthritis, it was found that intra-clinician consistency was excellent but inter-clinician consistency was moderate (Tragord et al., 2013). In general, standardized, quantified protocols would provide not only useful guidelines for the application as well as the prescription of optimal dosage of manual therapy and thereby increase its effectiveness. As such, development of standardized, quantifiable protocols provides a first step for standards of practice, upholding treatment and biomechanical interventions across the field and its practitioners, while also facilitating the opportunity for individualized and tailored treatment.

Taken together, although therapeutic treatment will need to be tailored to individuals' joint pathology and pain, we hypothesize that an understanding of both exercise and manual therapy based on joint loading biomechanics, e.g., joint torques and force vectors, will benefit healing. It will also lead to the development of standardized protocols that potentiate healthy mechanobiological adaptation to promote musculoskeletal health throughout life.

## Linking joint torques to tissue and cell mechanotransduction

Dynamic loading and joint torques from physical movement, such as those imbued via exercise therapy and manual therapy, vary in direct relation to internal joint loading (Anderson et al., 2007). Gait analysis (Block and Shakoor, 2010; Dorn et al., 2015) and joint torque modeling (Anderson et al., 2007) show that internal joint loads can be predicted consistently in relation to external joint torques. Hence, external torques are “internalized” at joints and physical therapy can be used prospectively to achieve target joint loading patterns with the goal to modulate the biomechanical environment of the joint (organ) and its constituent tissues. The physical linking of tissue compartments in the joint results in sharing and dynamic coupling of intersegmental forces passing through a given joint. Joints are complex, composite musculoskeletal structures made up of tissues of varying mechanical and biological properties that interact. Thus, once internalized, external forces are transferred through various tissue structures and attachments. Here we focus on how internalized forces are transferred through relevant musculoskeletal structures at both the tissue and cellular levels. *In addition, the remainder of this narrative review discusses published work specifically relevant to musculoskeletal force transfer and mechano- transduction and -adaptation*.

### Force transfer at the tissue level

Biological tissues inherently span multi-length scales through their hierarchical structure and function. The body is comprised of four tissues (muscle, connective, epithelial, and nervous) of varying mechanical properties and tissue architectures. As the main structural components of the musculoskeletal system, muscles, and connective tissues are particularly relevant for tissue-level mechanotransduction. The mechanical properties of muscle and connective tissues are dependent on their cellular and extracellular matrix components, including interstitial fluid (Culav et al., 1999; Knothe Tate, 2003).

#### The muscle-tendon unit

The muscle-tendon unit (MTU) actuates joint torque and hence movement. Muscle is the greatest contributor to joint support, stability, force generation, forward propulsion, and shock absorbance (Anderson and Pandy, 2003; Liu et al., 2008; Hamner et al., 2010; Franz and Kram, 2012, Figures 3C, 4A,B). The MTU spans the muscle, tendon and tendon enthesis, and its mechanical capacity and ability to transfer force is borne out of its hierarchical architecture (Purslow, 2010). In muscle tissue, forces (such as those to propagate and support physical movement) are generated through myocyte contraction and relaxation.

Muscle forces are transmitted through the muscle fascia, a hierarchical connective tissue consisting of contiguous enveloping membranes woven of collagen and elastin fibers. Collagen fibers are abundantly synthesized proteins that maintain structural integrity of the tissue through their high resistance to tensile loads (Culav et al., 1999; Viguet-Carrin et al., 2006). The hierarchical structure of the fascia membrane spans from the epimysium that encapsulates the whole muscle belly to the perimysium that surrounds each fascicular bundle in a collagen fiber weave, and then to the endomysium that envelopes individual fascicles. These layers, although described in hierarchical terms, together form a complex mesh comprising intertwining collagen fibers to allow for contiguous force distribution (Purslow, 2010). The crimped collagen fibers within fascial membranes enable storage of energy within and provide an organic physical manifestation of a spring/damper system. Under axial tension, collagen fibers, though normally crimped, begin to “uncramp,” albeit with minimal elongation (<10%). To this extent, collagen fiber arrangement and alignment is reflective of the mechanical environment of the tissue (Culav et al., 1999).

#### Bones

As connective tissues that are part of the musculoskeletal system, bones are loaded through direct contact with joint intersegmental forces (Zajac et al., 2002; Anderson and Pandy, 2003) and muscle contractions (Suominen, 2006, Figure 4C). During dynamic loading, compressive and tensile forces result in torsion and bending moments, particularly in long bones such as the femur, where forces are distributed from the femoral head to the shaft, concentrating at the femoral neck and midshaft. Bones are able to withstand large compressive and tensile loads due to their architecture including dense cortical and spongy trabecular bone matrix organization. Cortical bone correlates to regions of stress concentration, which reflects in the mineralized microstructure and protein network of the lamellar (onion ring like layers) network of cortical bone (Rho et al., 1998; Weiner and Wagner, 1998; Viguet-Carrin et al., 2006). Conversely, the mesh-like organization of trabecular bone forms a material and architecture conducive to flexibility and absorption of impact loads (Mente and Lewis, 1989).

Previous studies have shown pericellular fluid flow to be an important means for indirect force transfer and mechanotransduction in bone. Under dynamic strain, the hierarchical network of fluid-filled spaces interspersed throughout bone allows for pericellular fluid flow, causing fluid dynamic-associated pressure changes. Moreover, fluid is preferentially driven through regions with the greatest net change in strain, resulting in nonlinear mass transport and triggering of subcellular signaling pathways (Piekarski and Munro, 1977; Tate et al., 2000; Cowin, 2002; Zernicke et al., 2006; Anderson and Knothe Tate, 2008; McBride et al., 2011). Through fluid flow, hydrostatic forces have also been reported to transfer through the sub-periosteal space between the bone and the periosteum (Pitkin et al., 2015) and the permeability of periosteum itself exhibits direction- and flow rate dependent permeability (Evans et al., 2013). Taken together, bone can be described as both a poroelastic material which channels and harnesses mechanically induced fluid flow for mechano-chemical transduction and associated re-/modeling processes (Knothe Tate, 2003) as well as an organ that mediates the transfer of forces and mechanical load modulated mass transport from the joint level through to the cellular levels (Tate and Niederer, 1998).

Thus, the hierarchical structure and architecture of the musculoskeletal system provides a means for force transfer through multi-length scales at the tissue level. These structures are bound to each other through connective tissues, e.g., continuous collagen fibers of muscle fascia spanning the periosteum and penetrating as Sharpey's fibers into cortical bone (Figures 4D,E). The connective tissues are comprised of collagen fibers that resist tension under loading and confer forces between neighboring tissue structures (Carpenter and Carter, 2008; Moore et al., 2014a,b). Therefore, due to the interconnectedness of joint tissue structures, dynamic loading caused by joint torques are distributed throughout the joint and transferred to surrounding musculoskeletal components.

### Force transfer at the cellular level

At the cellular level, joint intersegmental forces are ultimately sensed by the mechanosensitive cells residing within musculoskeletal tissues. At this length scale, tissue stress-strains present as dilatational (e.g., due to hydrostatic forces) and/or deviatoric stresses on cells (e.g., due to shear forces, Knothe Tate, 2003; Anderson and Knothe Tate, 2007; Knothe Tate et al., 2008, 2016a). However, in reality cells within the body never experience purely dilatational or deviatoric forces as the local environment of the cell is mechanically complex and dynamic. For the purposes of linking joint therapy to cellular mechanotransduction, the mechanical milieu of the cell may be taken to be in context of dominant mechanical forces in the local environment, which may be impacted significantly by global force transduction.

#### Focal adhesions, cell cytoskeleton, and cell surface receptors

Mechanosensitive cells are able sense local strains through focal adhesions, cytoskeletal tensegrity, and cell surface receptors. Focal adhesions are mechanosensitive protein structures that resist tension developed in the cell architecture (Fouchard et al., 2014) and enable cells to mechanically couple and exert traction onto the extracellular matrix (ECM) (Lamothe et al., 2005; Stricker et al., 2011). The cell cytoskeleton is comprised of filaments that are tough and resilient to tension (actin) and compression (tubulin). Similarly to focal adhesions, the cytoskeleton allows cells to resist deformation under mechanical stimulation (Zimmermann and Tate, 2011; Knothe Tate et al., 2016a), which influences the overall shape and stiffness of cells (Jalilian et al., 2015). The significance here is that cell shape and architecture affects cellular mechanotransduction and downstream cell signaling and differentiation (McBeath et al., 2004; McBride and Knothe Tate, 2008; McBride et al., 2008; Chang and Tate, 2011). Cell surface receptors respond to force-induced conformational changes on the cell membrane. Stretch-activated channels are an example of cell surface receptors that mediate mechanosensitivity through biochemical processes and downstream cell signaling pathways, which in turn are capable of altering cell behavior and protein synthesis (Ingber, 2008; Servin-Vences et al., 2017).

#### Mechanosensitive cells

Mechanosensitive cells are abundantly present in all tissues of the body. Three examples of mechanosensitive cells that reside in and or transit through musculoskeletal tissues are the mesenchymal stem cell (MSC), the osteocyte and the red blood cell (RBC). These cells in particular provide an interesting study in contrasts, e.g., between the unspecialized cell which itself serves as a sensor and actuator of mechanical stimuli and mechanoadaptation, respectively, and the ultra specialized adherent (osteocyte) and perfectly motile (RBC) cell exhibiting specialized structures to transduce mechanical stimuli (Knothe Tate et al., 2016a).

Mesenchymal cells show three orders of magnitude more sensitivity to stress (circa 0.02 Pa) than terminally differentiated (mature) cells, such as osteocytes and chondrocytes (circa 1–2 kPa) (McBride et al., 2008; Chang and Tate, 2011). MSCs residing in musculoskeletal tissues can sense mechanical stimuli through direct interaction with their surrounding extracellular matrix (ECM) and their own cytoskeleton. This is moderated through various intracellular signaling pathways, e.g., the mitogen-activated protein kinase pathway that alters focal adhesion efficacy and cell adhesion to surrounding ECM (Mathieu and Loboa, 2012), and mechanically-gated ion channels in the cell membrane (Servin-Vences et al., 2017).

Osteocytes are the primary mechanosensors of bone (Burger and Klein-Nulend, 1999; Huang and Ogawa, 2012). They are situated within fluid-filled lacunae and, through their processes which extend through channels called canaliculi, form an interconnected network between other cells including osteocytes, osteoblasts, and cells of the periosteal and bone marrow niche (Temiyasathit and Jacobs, 2010; Tate and Fath, 2016; Tate et al., 2016). Situated within fluid-filled canaliculi, osteocytes directly sense the respective dynamic hydrostatic pressure and fluid shear on their cell bodies and processes (Anderson et al., 2005; Wang et al., 2007). These pericellular flow fields are directly modulated by the mineralisation state and associated nano-/microanatomy of the pericellular space bounded by the mineralized matrix and the cell processes (Anderson and Knothe Tate, 2008, Figures 4F,G). The fluid drag forces thus imparted on the surfaces of the osteocyte processes and the osteocyte bodies thus generate both deviatoric as well as dilatational stresses on osteocytes (Knothe Tate, 2003; Anderson et al., 2005) and are modulated over time by re-/modeling of the ECM and its mineralization. Also, fluid flow induces osteocytes to stretch and strain in the direction of the flow, causing a change in the geometry and architecture of the cell itself (Chang and Tate, 2011; Klein-Nulend et al., 2012).

Therefore, regarding mechanotransduction at the cellular level, cells sense dilatational and deviatoric forces on their surfaces, which causes the cell to change shape and size. These deformations are sensed by and transduced between cells through interaction of cellular components (focal adhesions, cell cytoskeleton, stretch-activated channels) and the extracellular matrix. The organization of the actin, tubulin, and intermediate fibers between the cell membrane and the nuclear membrane itself exhibits anisotropy, which provides a further means by which shape and volume changing stresses can direct changes in nuclear shape and volume (McBride et al., 2008). Taken together, through signaling pathways and the cell cytoskeleton, forces are transduced to the cell nucleus, where protein transcription is directly and indirectly modulated by conformational changes in the nucleus. Up- and down regulation of structural proteins making up the ECM also modulates downstream stress and strain sensed at the cell level, through changing of the mechanical *milieu* of the cell (Knothe Tate et al., 2016a).

## Molecular mechanisms of multi-scale mechanoadaptation via cell-mediated tissue re-/modeling

Adaptation and remodeling, at both cellular and tissue levels, encompass the mechanobiological response of cells, the living inhabitants of tissues, to mechanical loading. Dilatational and deviatoric stresses modulate cell differentiation, where lineage commitment type depends on the magnitude and direction of stresses and strain incurred locally by global physical activity (Waddington, 1957; Thompson, 1961; Pauwels, 1976; Prendergast et al., 1997; Carter et al., 1998; Stella et al., 2010; Song et al., 2013, Figure 1). Recent paired experimental and computational studies have begun to “map the mechanome,” the mechanics equivalent of the genome which provides a reference library of dilatational and deviatoric stresses conducive to achieve targeted lineage commitment (Anderson and Knothe Tate, 2007; Song et al., 2010, 2012, 2013; Knothe Tate et al., 2016a). In addition, cells up- and/or down-regulate the transcription of ECM structural proteins such as collagen, elastin, and proteoglycans, in response to mechanical stimulation (Ng et al., 2017). Hence, the composition and architecture of cell-generated ECM not only reflects the cell's mechanical history, but also literally forms its local environment and thereby influences its future mechanical loading history, throughout the life cycle of the cell (Knothe Tate et al., 2016a).

Throughout life, cells build and remodel the ECM that constitutes their local environment and which, in the case of connective tissues, essentially comprises a collagen and elastin fiber mesh. Such meshes are self assembled and adapted in response to the inhabitant cells' mechano-chemo-biological world. Over time, the ECM reflects the life history of its inhabitant cells (Knothe Tate et al., 2016a), integrating gene transcription, protein synthesis, post-translational modification, and cellular remodeling (e.g., osteoclastic resorption followed by osteoblastic osteoid deposition in the case of bone) in response to fluctuations in mechanical and mechanically modulated biochemical stimuli. As cells create their extracellular matrix, they are also simultaneously re/modeling the architecture and mechanical properties of their local environment and tissue structure. Hence, the architecture of ECM is constantly adapting in cognizance of its mechanical *milieu*, and the cell itself modulates its own local environment. In the past two decades there has been considerable interest in determining how cellular mechanobiology drives tissue mechanoadaptation during dynamic loading. The recent studies noted above will pave the way to proactive prescription of mechanical stimuli for targeted molecular and cell-mediated tissue mechanoadaptation.

## Mechanotherapy driven mechanoadaptation at the systemic length scale

All physical movements impart mechanical forces on the body. Physical therapy, including exercise therapy and manual therapy, are means in which mechanical stimuli can be used to invoke tissue regeneration, re/modeling or repair, i.e., mechanotherapy (Huang et al., 2013; Thompson et al., 2016). As forms of mechanotherapy, joint loading, low-level vibrations, and dynamic hydraulic stimulation are loading modalities that are being used for therapeutic benefit (see below). The ultimate aim of these technologies is to stimulate the body through mechanical intervention replicative of exercise in order to drive anabolic tissue adaptation toward healing (Figure 2). Another novel approach is extracorporeal shockwave therapy, which has been used as an exogenous means to induce tissue healing and remodeling in a variety of musculoskeletal contexts. These technologies are designed to be non-invasive and have been applied predominantly to the tissues of the musculoskeletal system including bones, joints and tendon/ligament insertions.

### Joint loading

Joint loading is a form of mechanotherapy that is commonly encountered in physical therapy treatment, e.g., during prescriptive exercise and manual therapy or mobilizations. As mentioned, forces interacting at joints are representative of the total load that is passing through the joint via intersegmental forces and dynamic coupling. The mechanism of action of joint loading can be explained by the anabolic and catabolic capacities of the body's joints; these capacities arise i.e., from the joint's capability to shift the intramedullary pressure within the intramedullary cavity along the length of the limb during cyclic alteration (Zhang et al., 2007) as well as cross talk between tissue compartments within the joint. At smaller length scales, joint loading modulates interstitial molecular transport driven by both strain and pressure gradients, thus creating not only chemical gradients but also streaming potentials that promote tissue remodeling within each respective compartment (Zhang et al., 2007, 2008; Freutel et al., 2014; Knothe Tate et al., 2016b). Previous work has demonstrated knee loading to be a potent mechanical stimulus for bone mineral deposition. Minute strains, i.e., <20 microstrain, expedite the closure of surgical wounds, facilitate callus formation, and significantly increase tibial and femoral cortical thickness (Zhang et al., 2007). Thus, joint loading can be seen as an effective method to mechanically stimulate healing and tissue regeneration.

### Low magnitude mechanical signals

Low magnitude mechanical signals (LMMS), whether imbued via vibrations or oscillations, increase the anabolic potential of bone tissue. This mode of mechanotherapy has been shown to increase both trabecular and cortical bone mass density in patients with bone fractures after 6–12 months of treatment. The same studies combined low magnitude with high frequency loading, as tissues are biologically sensitive to high frequency events. Low magnitude-high frequency (LMHF) stimuli improved trabecular bone mass density by 34% (Ozcivici et al., 2010a,b). Hence, LMMS and LMHF may provide promising means to induce anabolic processes through mechanical stimulation. As with ESWT, additional clinical trials are needed.

### Dynamic hydraulic stimulation

Dynamic hydraulic stimulation (DHS) provides a non-invasive technique to deliver dynamic pressure to tissues of the skin and muscle. In a rat model of functional disuse, DHS was used, alternating 30 mmHg of static and 30 mmHg of dynamic, DHS was used, alternating 30 mmHg of static and 30 mmHg of dynamic loading to the muscle tissue surrounding the tibiae. Following this loading regimen, DHS stimulated tibiae exhibited an 83% increase in bone volume and 190% greater bone apposition rate in comparison to their non-stimulated counterparts (Hu et al., 2012; Yokota et al., 2012). DHS has the potential to promote anabolic activity recapitulating mechanisms of joint loading regimens, in which mechanical stimuli applied to limbs are able to direct intramedullary fluid flow and pressure, and in turn elicit a mechanobiological response.

### Pulsed electromagnetic fields and extracorporeal shock wave therapy

Due to their capacity to promote natural healing pathways, exogenous biophysical therapies such as pulsed electromagnetic fields (PEMF) and extracorporeal shock wave therapy (ESWT) have garnered much interest of late. These therapies play a putative role in the modulation of natural inflammatory pathways, growth factor upregulation and protein synthesis (Selvamurugan et al., 2007; Petecchia et al., 2015; Rosso et al., 2015; D'agostino et al., 2015; Zhai et al., 2016). Although the body of literature describing the empirical use of PEMF for treatment of bone and joint injuries spans three decades, recently PEMF has gained interest for the promotion of muscle quality and tendon-to-bone healing after surgical repair (Liu et al., 2017). Similarly, ESWT, also referred to as lithotripsy, has been used increasingly to treat a number of sports injuries (e.g., tendinopathies and plantar fasciopathy) as well as disorders in bone regeneration and chronic wounds in both soft and hard tissues (Vahdatpour et al., 2012; Kisch et al., 2015; Rathleff and Thorborg, 2015; Rosso et al., 2015).

While studies are ongoing to determine the exact mechanisms underpinning ESWT's healing effects, animal studies implicate repair mechanisms stemming from the creation of controlled tissue damage, i.e., exogenous induction of microdamage or “wear and tear,” which mimics that occurring naturally through exercise and physical activity (Knothe Tate et al., 2013). Microdamage accumulates in bone in response to repeated, cyclic loading, also referred to as fatigue loading, and provides a powerful trigger for endogenous healing activity (Tami et al., 2003). While both PEMF and ESWT therapies show promise for the potentiation of endogenous healing mechanisms, controlled clinical trials are needed to test the efficacy and optimal dosing regimens for such treatments (Elsisi et al., 2015; Handoll et al., 2015; Hannemann et al., 2015; Rosso et al., 2015; Reilingh et al., 2016).

Hence, the principles and molecular mechanisms underpinning mechanotherapeutic therapies and technologies can be applied prospectively, at a systemic biomechanics level, to influence tissue remodeling and healing. Many of these technologies have been successfully applied to bone, and their application to modulate remodeling of the entire joint appears to be a logical next step. Thus, physical therapy techniques and treatments can be perceived as a form of mechanotherapy, since it involves mechanical or movement-related interventions to restore and optimize function. Since mechanical stimuli have been shown to act on all length scales of the body, the working hypothesis is that physical movements exert similar multiscale effects. If so, novel physical therapy regimens and other treatment modalities can be established to enhance healing and rehabilitation outcomes.

## Conclusions

The human body is an integrated, dynamic system in which forces are continuously transferred through joints, tissues and cells. At the same time, cells are constantly remodeling their biological milieu to adapt to their ever-changing mechanical environment. Because of this, mechanical stimulation is a potent regulator of cellular metabolism (anabolic and catabolic activities) and the physical properties of their synthesized matrix, which are manifested as tissue structures. Theoretically, mechanical stimuli can be “controlled” at physiological levels that encourage and stimulate constructive tissue adaptation and remodeling through engineering of physical therapy and exercise protocols. By directing loading regimens to specific sites, and having an understanding of the mechanotransduction and mechanobiological response across these length and time scales, physical therapy can be used to expedite healing and rehabilitation throughout life. In the future, exercise therapies, manual mobilizations and other physical modalities used in physical therapy may be tailored to facilitate (i) the recruitment, migration and targeted lineage commitment of stem cells, (ii) expedited tissue genesis, and (iii) optimal remodeling such that healed tissues meet or exceed their native mechanical strength.

## Author contributions

This article was written and edited by the coauthors, JN, MK, SK, and MKT.

### Conflict of interest statement

The authors declare that the research was conducted in the absence of any commercial or financial relationships that could be construed as a potential conflict of interest.

## Footnotes

^1^Here and throughout the manuscript, *re-/modeling* refers to modeling and/or remodeling of tissues by cells. Specifically, *remodeling* describes the cycle by which bone maintains a dynamic steady state through osteoclastic resorption followed by osteoblastic deposition of bone at the same site. In contrast, modeling describes the apposition of new bone by osteoblasts, resulting in changes to the size and shape of the bone.
